# A prospective study of the relationship between illness perception, depression, anxiety, and quality of life in hematopoietic stem cell transplant patients

**DOI:** 10.1002/cam4.6906

**Published:** 2024-01-11

**Authors:** Steven C. Ames, Lori Lange, Gretchen E. Ames, Michael G. Heckman, Launia J. White, Vivek Roy, James M. Foran

**Affiliations:** ^1^ Division of Hematology and Oncology Mayo Clinic Florida Jacksonville Florida USA; ^2^ Department of Psychology University of North Florida Jacksonville Florida USA; ^3^ Department of Psychiatry and Psychology Mayo Clinic Florida Jacksonville Florida USA; ^4^ Division of Clinical Trials and Biostatistics Mayo Clinic Florida Jacksonville Florida USA

**Keywords:** behavioral science, cancer stem cells, QOL, quality of life

## Abstract

**Aim:**

The aim of study was to investigate whether depression and anxiety symptoms and illness perception prior to hematopoietic stem cell transplantation (HSCT) predict health related quality of life (HRQOL) at Day 100 and 1 year following HSCT.

**Methods:**

A total of 205 patients who underwent HSCT (*N* = 127 autologous transplants, *N* = 78 allogeneic transplants) were included in this prospective study. Baseline assessment was assessed prior to transplantation and post HSCT data were collected at Day 100 and 1 year. At baseline we assessed depressive symptoms (Patient Health Questionnaire‐9), anxiety symptoms (Generalized Anxiety Disorder‐7), illness perception (Brief Illness Perception Questionnaire), and HRQOL (Functional Assessment of Cancer Therapy‐BMT).

**Results:**

Patients who expressed a greater level of concern about the severity, course, and ability to exert control over one's illness (i.e., illness perception) and who reported a greater level of depression and anxiety symptoms prior to HSCT reported lower HRQOL at both Day 100 and 1 year posttransplant, with a similar degree of association observed at the two follow‐up time points.

**Conclusions:**

Our findings suggest that pretransplant perceptions about their illness and negative mood are significant predictors of HRQOL following HSCT. Illness perception, depression, and anxiety are potentially modifiable risk factors for less than optimal outcome after HCSCT and intervention strategies should be explored.

## Graphical abstract

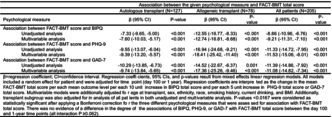

## 1 INTRODUCTION

Hematopoietic stem cell transplantation (HSCT) is a complex medical intervention used to treat a variety of hematological disorders and other malignant conditions.^1^, ^2^ While HSCT offers effective disease control or potential cure for hematopoietic malignancies, the experience is demanding for the patient.^3^, ^4^ Patient demands include 3–4 weeks of hospitalization, a prolonged period of isolation and physical recovery posttransplantation, and significant risk from complications.^1^ Complications may include chronic graft versus host disease, cardiopulmonary compromise, endocrinopathies, musculoskeletal disorders, and subsequent malignancies.^5^, ^6^ Collectively, the uncertainty about success and risk of morbidity and mortality is recognized as a significant source of stress for HSCT patients and their caregivers.^3^, ^4^, ^7^ Various aspects of quality of life, including health related quality of life (HRQOL), has been found to be diminished during hospitalization for HSCT with improvement occurring over the months following discharge.^8^, ^9^, ^10^

The concept of illness perception arose out of Leventhal's self‐regulation theory^11^ and proposes that cognitive representations of illness influence behavioral responses and coping. These cognitive representations include beliefs about cause, severity, course, and ability to exert control over one's own illness. Illness perception have been found to influence health practices and psychological distress in a wide range of illnesses.^12^, ^13^ To our knowledge, illness perception in HSCT has been investigated in only one prior study. This study found that HSCT recipients who perceived the consequences of their cancer to be more serious experienced more depression, anxiety, and reduced well‐being during the year following transplant.^14^ Whether illness perception impacts the HRQOL of HSCT patients has not been studied. Information about whether illness perception predicts HRQOL following HSCT has implications both for pretransplant psychosocial assessment and for developing intervention approaches to optimize HSCT outcomes.

Mental health factors including depression and anxiety have been found to be negatively associated with quality of life prior to HSCT.^15^ Studies that have assessed the trajectory of distress and quality of life posttransplant have found that patients report the highest levels of anxiety and/or depression during hospitalization or at discharge.^16^, ^17^, ^18^ Emotional functioning which included feelings of sadness and anxiety at the time of HSCT was found to predict quality of life 1 year post HSCT.^19^ Our study adds to the existing literature by investigating not only depression and anxiety symptoms, but also how illness perception may influence HRQOL at both Day 100 and 1 year post HSCT. The Day 100 and 1 year time points are particular interest as prior studies have suggested that mental health factors tend to improve by 100 days post transplant^17^, ^20^ while HRQOL tends to improve by 1 year post transplant.^21^

The aim of study was to investigate whether depression and anxiety symptoms and illness perception prior to HSCT predict HRQOL at Day 100 and 1 year following HSCT. Based on theoretical and prior empirical findings, we hypothesized that a greater level of depression and anxiety symptoms and having a more threatening perception of illness at baseline assessment prior to HSCT will predict diminished HSCT at both Day 100 and 1 year post transplant.

## 2 METHODS

### 2.1 Patients and data collection

Patients eligible for this study were adults ≥18 years of age undergoing autologous or allogeneic HSCT. Inclusion criteria included the ability to speak and read English. The individuals who participated in this investigation provided written informed consent. All HSCT patients receive a comprehensive multi‐specialty assessment prior to transplantation and baseline assessment was completed in person during this period of time. Information was collected regarding baseline characteristics (age at transplant, sex, ethnicity, race, marital status, level of school completed, current employment status, approximate annual household gross income, smoking history, primary disease type, current alcohol consumption, and BMI), psychological factors (Brief Illness Perception Questionnaire [BIPQ], Patient Health Questionnaire‐9 [PHQ‐9], Generalized Anxiety Disorder‐7 [GAD‐7]), and outcomes (Functional Assessment of Cancer Therapy‐BMT [FACT‐BMT] at Days 100 and 1 year following BMT). Results are presented separately for the autologous transplant patients, allogeneic transplant patients, and the overall patient group. Post HSCT data were collected at Day 100 and 1 year primarily via mail, or in person when possible. All study measures were administered using paper versions. This study was approved by the Mayo Clinic institutional review board (IRB study ID: 14‐004628).

### 2.2 Psychological and health‐related quality of life assessments

#### 2.2.1 Depression symptoms

The PHQ‐9^22^ is 9‐Item reliable and valid self‐report measure of depression symptoms that is widely used as a screening tool in health care settings.^23^ Patients answer items on a Likert scale from 0 (not at all) to 3 (nearly every day). On this measure responses can be categorized according to level of depression including minimal (0–4), mild (5–9), moderate (10–14), moderately severe (15–19), and severe (20–27). The PHQ‐9 was administered at baseline, Day 100, and 1 year.

#### 2.2.2 Anxiety symptoms

The GAD‐7^24^ is a reliable and valid 7‐item self‐report measure of anxiety symptoms that is widely used as a screening tool in health care settings.^25^ Patients answer items on a Likert scale from 0 (not at all) to 3 (nearly every day). On this measure responses can be categorized according to level of anxiety including minimal (0–4), mild (5–9), moderate (10–14), and severe (15–21). The GAD‐7 was administered at baseline, Day 100, and 1 year.

#### 2.2.3 Illness perception

The BIPQ^26^ items pertain to illness perception (i.e., consequences, timeline, personal control, treatment control, identity, coherence, and emotional representation). Examples of items include: How much control do you feel you have over your illness?: 0 (absolutely no control) to 10 (extreme amount of control); How much do you think your treatment can help your illness?: 0 (not at all) to 10 (extremely helpful); How well do you feel you understand your illness?: 0 (don't understand at all) to 10 (understand very clearly). Items are answered on a Likert scale from 0 to 10 and response anchors differ for each item depending on the nature of the question asked as described above. Items were summed, with certain items being reverse‐scored, to obtain an overall score (range: 0–80), with higher scores her scores on this measure reflecting a more threatening view of the illness. The BIPQ has been found to be both a reliable and valid measure of the cognitive and emotional representations of illness.^26^ Previous research has demonstrated that illness perception is generally stable over longer periods of time (i.e., 2 years)^27^, ^28^ therefore, similar to a prior study in patients undergoing HSCT^14^ we measured illness perception at baseline.

#### 2.2.4 Health related quality of life

The FACT‐BMT^29^ was administered at Day 100 and 1 year post HSCT to assess HRQOL. The FACT‐BMT is a reliable and validated measure that is commonly used to assess stem cell transplant specific quality of life in clinical trials.^29^ The questions are rated on a four‐point Likert scale from 0 (not at all) to 4 (very much). Certain items are reverse‐scored so that a higher summed score reflects better functioning (range: 0–148).

### 2.3 Statistical analysis

Missing data for the individual questions used to calculate the BIPQ, PHQ‐9, GAD‐7, and FACT‐BMT total scores was imputed as follows. For patients with missing data for more than 20% of the individual questions for a given measure, the total score was considered to be missing. For patients with missing data for less than 20% of the individual questions for a given measure, missing values for individual questions were imputed using the average value of the patients who answered the given question. Imputation was utilized for individual questions only and was not used for total scores of BIPQ, PHQ‐9, GAD‐7, and FACT‐BMT.

The extent of imputation of missing data was generally minimal. Using the aforementioned missing data imputation strategy, for baseline BIPQ, 202 patients did not require imputation, 1 patient had data imputed (only 1 question was imputed), and 2 patients were considered as missing. Regarding baseline PHQ‐9, 200 patients did not need imputation and 5 patients had data imputed (all 5 only had imputation for 1 question). The baseline GAD‐7 did not require any imputation for 201 patients, with the remaining 4 patients having data imputed (all 4 only had imputation for 1 question). For the FACT‐BMT, at Day 100 a total of 97 patients did not need any imputation, 57 had data imputed (Median number of questions imputed = 1), and 51 patients were considered as missing, while at 1 year 90 patients did not require any imputation, 49 had data imputed (Median number of questions imputed = 1), and 66 patients were considered as missing. Given the greater extent of missing data for the FACT‐BMT at follow‐up time points compared to baseline BIPQ, PHQ‐9, and GAD‐7, in Table S1 we have displayed the number of FACT‐BMT questions answered (of 37 total) at the Day 100 and 1‐year time points.

Continuous variables were summarized with the sample median and range. Categorical variables were summarized with number and percentage of patients. Comparisons of characteristics between patients with and without FACT‐BMT information available were made using a Wilcoxon rank sum test (continuous and ordinal variables) or Fisher's exact test (categorical variables). Comparisons of PHQ‐9 total score and GAD‐7 total score between baseline, Day 100, and 1 year time points were made using a paired Wilcoxon signed rank test; *p*‐values <0.0167 (i.e., 0.05/3) were considered as statistically significant after applying a Bonferroni correction for multiple testing for the three pair‐wise comparisons made for each psychological measure. Pair‐wise correlations between baseline PHQ‐9 total score, baseline GAD‐7 total score, and baseline BIPQ total score were assessed using Spearman's test of correlation.

Associations of baseline PHQ‐9 total score, baseline GAD‐7 total score, and baseline BIPQ total score with FACT‐BMT (in the autologous transplant subgroup, the allogeneic transplant subgroup, and in all patients) were evaluated using unadjusted and multivariable mixed effects linear regression models. All models included a random effect for patient and were adjusted for time point (Day 100 or 1 year). Multivariable models were considered as the primary analysis and were additionally adjusted for the pre‐defined potential confounding variables of age at transplant, sex, ethnicity, race, smoking history, current drinking, and BMI. Additionally, transplant subgroup was also adjusted for in analysis of all patients in both unadjusted and multivariable analysis. Presence of interactions of time point with PHQ‐9 total score, baseline GAD‐7 total score, and baseline BIPQ total score were also assessed. Regression coefficients (referred to as *β*) and 95% confidence intervals (CIs) were estimated and are interpreted as the increase in the mean FACT‐BMT total score corresponding to a specified increase in the given psychological factor. We utilized a Bonferroni correction for multiple testing in order to account for the three different psychological measures that were assessed for association with FACT‐BMT total score, after which *p*‐values <0.0167 (i.e., 0.05/3) were considered as statistically significant. All statistical tests were two‐sided. Statistical analyses were performed using SAS (version 9.4; SAS Institute, Inc., Cary, North Carolina).

## 3 RESULTS

A total of 205 patients who underwent HSCT (*N* = 127 autologous transplants, *N* = 78 allogeneic transplants) at the Mayo Clinic in Jacksonville, Florida between December 2014 and October 2020 were included in this prospective study. A total of 83 individuals declined an invitation to participate in this study, resulting in a response rate of 71.2%. Participant characteristics at baseline are summarized in Table 1, while patient scores on psychological and HRQOL measures are displayed in Table 2. The BIPQ, PHQ‐9, GAD‐7, and FACT‐BMT are further illustrated in Figure S1 for the separate autologous and allogeneic transplant groups. A summary of participant responses to the individual items of the BIPQ is presented in Table S2. Of note, when comparing characteristics between the 40 patients without FACT‐BMT data available at Day 100 or 1‐year to the 165 patients with such data available (Table S3), no differences were noted except for primary disease type (*p* < 0.001) and type of transplant (*p* = 0.002). Of note, BIPQ total score (*p* = 0.78), PHQ‐9 total score (*p* = 0.85), and GAD‐7 total score (*p* = 0.74) were similar between the two groups.

**TABLE 1:** Patient characteristics.

	Autologous transplant (*N* = 127)		Allogeneic transplant (*N* = 78)		All patients (*N* = 205)	
Variable	*N*	Median (minimum, maximum) or no. (%) of patients	*N*	Median (minimum, maximum) or no. (%) of patients	*N*	Median (minimum, maximum) or no. (%) of patients
Age at transplant (years)	127	60 (22, 80)	78	59 (22, 74)	205	60 (22, 80)
Sex (male)	127	68 (53.5%)	78	39 (50.0%)	205	107 (52.2%)
Ethnicity (Hispanic/Latino)	126	6 (4.8%)	78	5 (6.4%)	204	11 (5.4%)
Race	125	21 (16.8%)	78	5 (6.4%)	204	26 (12.8%)
White		104 (83.2%)		73 (93.6%)		177 (86.3%)
Black or African American		18 (14.4%)		3 (3.8%)		21 (10.2%)
Other		3 (2.4%)		2 (2.6%)		5 (2.4%)
Marital status	127		78		205	
Never married		7 (5.5%)		5 (6.4%)		12 (5.9%)
Currently married		96 (75.6%)		55 (70.5%)		151 (73.7%)
Separated		3 (2.4%)		1 (1.3%)		4 (2.0%)
Divorced		15 (11.8%)		8 (10.3%)		23 (11.2%)
Widowed		2 (1.6%)		5 (6.4%)		7 (3.4%)
Cohabitating with partner		4 (3.1%)		4 (5.1%)		8 (3.9%)
Level of school completed	127		77		204	
Some high school or less		7 (5.5%)		5 (6.5%)		12 (5.9%)
High school/GED		28 (22.0%)		15 (19.5%)		43 (21.1%)
Some college/associate/technical		32 (25.2%)		26 (33.8%)		58 (28.4%)
College graduate		37 (29.1%)		24 (31.2%)		61 (29.9%)
Graduate school		23 (18.1%)		7 (9.1%)		30 (14.7%)
Current employment status	127		78		205	
Employed		45 (35.4%)		35 (44.9%)		80 (39.0%)
On leave		16 (12.5%)		16 (20.5%)		32 (15.6%)
Retired		39 (30.7%)		16 (20.5%)		55 (26.8%)
Disabled		20 (15.7%)		5 (6.4%)		25 (12.2%)
Unemployed		7 (5.6%)		6 (7.8%)		13 (6.3%)
Annual household gross income	122		76		198	
Less than $20,000		9 (7.4%)		5 (6.6%)		14 (7.1%)
$20,000–$39,999		19 (15.6%)		9 (11.8%)		28 (14.1%)
$40,000–$59,999		18 (14.8%)		11 (14.5%)		29 (14.6%)
$60,000–$79,999		22 (18.0%)		17 (22.4%)		39 (19.7%)
$80,000–$99,999		22 (18.0%)		12 (15.8%)		34 (17.2%)
$100,000 or more		32 (26.2%)		22 (28.9%)		54 (27.3%)
Smoking history	127		77		204	
Never smoked		69 (54.3%)		42 (54.5%)		111 (54.4%)
Past smoker		53 (41.7%)		31 (40.3%)		84 (41.2%)
Current smoker		5 (3.9%)		4 (5.2%)		9 (4.4%)
Illicit drug use						
Any illicit drug use	127	7 (5.5%)	78	6 (7.7%)	205	13 (6.3%)
Marijuana	127	7 (5.5%)	78	5 (6.4%)	205	12 (5.9%)
Prescription drugs	127	0 (0.0%)	78	1 (1.3%)	205	1 (0.5%)
Primary disease type	127		78		205	
Acute lymphocytic leukemia		0 (0.0%)		12 (15.4%)		12 (5.9%)
Acute myeloid leukemia		0 (0.0%)		25 (32.1%)		25 (12.2%)
Other acute leukemia		0 (0.0%)		2 (2.6%)		2 (1.0%)
Chronic myelogenous leukemia		0 (0.0%)		5 (6.4%)		5 (2.4%)
Myelodysplastic syndrome/disorder		0 (0.0%)		22 (28.2%)		22 (10.7%)
Hodgkin's disease		8 (6.3%)		4 (5.1%)		12 (5.9%)
Non Hodgkins lymphoma		27 (21.3%)		3 (3.8%)		30 (14.6%)
Plasma cell disease		89 (70.1%)		1 (1.3%)		90 (43.9%)
Aplastic anemia		0 (0.0%)		1 (1.3%)		1 (0.5%)
Chronic lymphocytic leukemia		1 (0.8%)		1 (1.3%)		2 (1.0%)
Other		2 (1.6%)		2 (2.6%)		4 (2.0%)
Alcohol history	126		78		204	
Never		62 (49.2%)		34 (43.6%)		96 (47.1%)
Monthly or less		34 (27.0%)		24 (30.8%)		58 (28.4%)
2–4 times a month		14 (11.1%)		14 (17.9%)		28 (13.7%)
2–3 times a week		9 (7.1%)		4 (5.1%)		13 (6.4%)
4 or more times a week		7 (5.6%)		2 (2.6%)		9 (4.4%)
Body mass index	123	28.8 (0.0, 55.6)	77	28.1 (18.9, 47.0)	200	28.5 (0.0, 55.6)

**TABLE 2:** Patient scores on psychological and health related quality of life measures.

	Autologous transplant (*N* = 127)		Allogeneic transplant (*N* = 78)		All patients (*N* = 205)	
Variable	*N*	Median (minimum, maximum) or no. (%) of patients	*N*	Median (minimum, maximum) or no. (%) of patients	*N*	Median (minimum, maximum) or no. (%) of patients
BIPQ total score (baseline)	127	34 (6, 62)	76	36 (5, 59)	203	35 (5, 62)
PHQ‐9 total score (baseline)	127	3 (0, 21)	78	3 (0, 17)	205	3 (0, 21)
GAD‐7 total score (baseline)	127	2 (0, 21)	78	2 (0, 19)	205	2 (0, 21)
FACT‐BMT total score						
Day 100	103	121.0 (81.7, 146.1)	51	111.1 (42.0, 136.0)	154	118.0 (42.0, 146.1)
1 year	95	122.0 (57.1, 147.0)	44	118.1 (42.0, 147.0)	139	122.0 (42.0, 147.0)

The PHQ‐9 and GAD‐7 were compared across time points and these results are presented in Table 3. No statistically significant (after multiple testing correction) differences between time points were identified. Of note, there were significant correlations between baseline PHQ‐9 total score and both baseline GAD‐7 total score (Spearman's *r*: 0.62, *p* < 0.001) and baseline BIPQ total score (Spearman's *r*: 0.45, *p* < 0.001), as well as between baseline GAD‐7 total score and baseline BIPQ total score (Spearman's *r*: 0.48, *p* < 0.001).

**TABLE 3:** Comparisons of PHQ‐9 total score and GAD‐7 total score across time points.

Time point	PHQ‐9 total score	GAD‐7 total score
Baseline		
*N*	205	205
Median (minimum, maximum)	3 (0, 21)	2 (0, 21)
Day 100		
*N*	156	156
Median (minimum, maximum)	3 (0, 23)	1 (0, 18)
1 year		
*N*	141	140
Median (minimum, maximum)	2.0 (0, 20)	1 (0, 21)
*p*‐value: Baseline vs. Day 100	0.90	0.30
*p*‐value: Baseline vs. 1 year	0.12	0.048
*p*‐value: Day 100 vs. 1 year	0.099	0.36

*Note*: *p*‐values result from a paired Wilcoxon signed rank test. *p*‐values <0.0167 were considered as statistically significant after applying a Bonferroni correction for multiple testing for the three pair‐wise comparisons that were made for each psychological measure.

Associations between psychological factors (i.e., BIPQ, PHQ‐9, and GAD‐7) with HRQOL at Day 100 and 1 year post HSCT are presented in Table 4. Results are presented separately for autologous and allogeneic transplants as well as in the combined group. In multivariable analysis adjusting for potential confounding variables and after correcting for multiple testing, there were significant associations of a greater BIPQ, PHQ‐9, and GAD‐7 scores with a lower FACT‐BMT total score at the two follow‐up time points in all three patient groups (all *p* < 0.001). There was no evidence of a difference in the degree of these associations between the Day 100 and 1‐year time points at which FACT‐BMT was measured (all interaction *p* ≥ 0.062). To illustrate further in the combined group of all patients where sample size is largest, in multivariable analysis FACT‐BMT was significantly associated with BIPQ (*β*: −9.21, *p* < 0.001), PHQ‐9 (*β*: −11.53, *p* < 0.001), and GAD‐7 (*β*: −11.08, *p* < 0.001).

**TABLE 4:** Associations of psychological factors at baseline with Day 100 and 1 year FACT‐BMT total score.

	Association between the given psychological measure and FACT‐BMT total score					
	Autologous transplant (*N* = 127)		Allogeneic transplant (*N* = 78)		All patients (*N* = 205)	
Psychological measure	*β* (95% CI)	*p*‐value	*β* (95% CI)	*p*‐value	*β* (95% CI)	*p*‐value
Association between FACT‐BMT score and BIPQ						
Unadjusted analysis	−7.33 (−9.65, −5.00)	<0.001	−12.55 (−16.77, −8.33)	<0.001	−8.86 (−10.96, −6.76)	<0.001
Multivariable analysis	−7.60 (−10.03, −5.17)	<0.001	−12.74 (−16.81, −8.68)	<0.001	−9.21 (−11.31, −7.10)	<0.001
Association between FACT‐BMT score and PHQ‐9						
Unadjusted analysis	−9.55 (−13.07, −6.04)	<0.001	−16.94 (−24.68, −9.21)	<0.001	−11.33 (−14.72, −7.95)	<0.001
Multivariable analysis	−9.39 (−13.20, −5.57)	<0.001	−18.41 (−25.42, −11.40)	<0.001	−11.53 (−15.06, −8.01)	<0.001
Association between FACT‐BMT score and GAD‐7						
Unadjusted analysis	−10.29 (−13.85, −6.73)	<0.001	−14.52 (−22.67, −6.37)	0.001	−11.39 (−14.86, −7.92)	<0.001
Multivariable analysis	−9.74 (−13.84, −5.65)	<0.001	−17.36 (−25.26, −9.46)	<0.001	−11.08 (−14.82, −7.34)	<0.001

Abbreviation: β = regression coefficient; CI = confidence interval.

*Note*: Regression coefficients, 95% CIs, and *p*‐values result from mixed effects linear regression models. All models included a random effect for patient and were adjusted for time point (Day 100 or 1 year). Regression coefficients are interpreted as the change in the mean FACT‐BMT total score per each mean outcome level per each 10 unit increase in BIPQ total score and per each 5 unit increase in PHQ‐9 total score or GAD‐7 total score. Multivariable models were additionally adjusted for age at transplant, sex, ethnicity, race, smoking history, current drinking, and BMI. Additionally, transplant subgroup was also adjusted for in analysis of all patients in both unadjusted and multivariable analysis. *p*‐values <0.0167 were considered as statistically significant after applying a Bonferroni correction for the three different psychological measures that were assessed for association with FACT‐BMT total score. There was no evidence of a difference in the degree of the associations of BIPQ, PHQ‐9, or GAD‐7 with FACT‐BMT total score between the Day 100 and 1‐year time points (all interaction *p* ≥ 0.062).

For interpretation, an increase of 10 points in the BIPQ at baseline predicted a 9.21 point mean reduction in the FACT‐BMT at follow‐up time points across the entire sample. Furthermore, 5‐point higher scores for the PHQ‐9 and GAD‐7 corresponded with 11.53 and 11.08 lower mean FACT‐BMT levels at Day 100 and 1 year. For the PHQ‐9, GAD‐7, and FACT‐BMT a 5‐point change in score is considered clinically significant.^23^, ^24^, ^29^ A score that would signify a substantial change in illness perception has not been established for the BIPQ.

## 4 DISCUSSION

This study evaluated the association of illness perception and depression and anxiety symptoms assessed prior to HSCT transplant with HRQOL at Day 100 and 1 year posttransplant. Consistent with our hypothesis, results show that pretransplant illness perception, depression symptoms, and anxiety symptoms were significant predictors of HRQOL post HSCT. Patients who expressed a higher level of concern about the severity, course, and ability to exert control over one's illness (i.e., illness perception) and who reported a higher levels of depression and anxiety symptoms prior to HSCT experienced lower HRQOL at both Day 100 and 1 year posttransplant. These findings were consistent in autologous and allogeneic patients as well as in the combined group that included all patients.

### 4.1 Clinical Implications

Our study is the first to evaluate whether pretransplant illness perception and depression and anxiety symptoms predict HRQOL at both Day 100 and 1 year following HSCT. This pattern of results for the association of depression and anxiety symptoms with HRQOL is consistent with prior investigations that assessed this relationship at other time points.^16^, ^30^ Our investigation suggests that patients preparing to undergo HSCT who express feeling of depression and anxiety would benefit from intervention efforts to address these concerns prior to transplant. While HRQOL following HSCT is clearly an important outcome on its own, studies have also found psychological factors including depression and anxiety associated with higher posttransplant hospital readmission,^31^ morbidity,^32^ and mortality.^33^, ^34^ Additionally, addressing depression and anxiety prior to and during recovery from HSCT could positively impact patient ability to engage in their recovery, adhere fully to treatment recommendations, and better cope with the inevitable uncertainty and multitude of potential neuropsychological and physical complications.^35^

Our findings also suggest that patients would benefit from additional efforts to assess and when appropriate enhance illness perception prior to HSCT. Some patients may have inaccurate negative perceptions about their illness and/or HSCT. For example, a given HSCT patient may express that they have a poor understanding of their illness or have little confidence that HSCT will be of benefit to them. These inaccurate perceptions may be readily addressed via additional education from the health care team. However, as our study was designed to study the association between the construct of illness perception and HRQOL and not the beliefs that influence illness perception future research is needed.

Since the median score on the PHQ‐9 and GAD‐7 was in the minimal symptom range, our findings suggest that HSCT patients with higher scores, particularly those scoring about the minimal symptom range with a scores of 5 or greater on either the PHQ‐9 or GAD‐7, be closely followed and potentially identified and offered additional mental health support. An additional consideration is that HSCT patients often have fewer medical appointments after Day 100, and thereby potentially access to mental health services are also diminished after this time point. Given our findings that depression and anxiety symptoms remain associated with diminished HRQOL at 1 year, individuals reporting increased depression or anxiety prior to transplant may benefit from continued mental health services beyond Day 100.

Only a few studies been undertaken to develop an intervention to address HRQOL in HSCT patients. One investigation found a positive psychology intervention to be feasible and well accepted by HSCT patients.^36^ Two other studies found that the addition of inpatient palliative care resulted in improved HRQOL and less depression following HSCT^37^ and lower depressive and post‐traumatic stress disorder symptoms.^38^ Thus, as both our findings and prior evidence are consistent in finding illness perception and depression and anxiety symptoms related to HRQOL following HSCT, additional work is clearly needed in this area.^39^ Research is also needed to examine the optimal time in relation to HSCT to deliver an intervention. If psychological wellness were improved, patients might experience improved HRQOL throughout the HSCT process which also has the potential to improve posttransplant medical outcomes.

### 4.2 Study limitations

Several limitations of this study are important to consider. First, the sample size is not overly large, and therefore, the possibility of a type II error (i.e., a false‐negative finding) is important to consider, particularly in the relatively small allogeneic transplant subgroup. Second, we cannot rule out the possibility that our results could have been influenced by variables that we did not adjust for in our multivariable models, or by unmeasured confounding variables. Third, the patients from our single‐center study represent a specific population (i.e., most were White with a greater than high school level of education), and therefore our findings may not be generalizable to other patient populations. Fourth, our study did not assess associations of depression, anxiety, and illness perception with clinical outcomes such as survival and relapse, as this was beyond the scope of our study. Nonetheless, this will be an important topic for future study. Finally, information regarding FACT‐BMT was unavailable at either follow‐up time point for approximately 20% of the study patients, which is not unexpected as this information was primarily collected via mail. However, other than differences regarding primary disease type, type of transplant, and to a lesser extent BMI, no major differences in patient characteristics were noted between patient with and without follow‐up FACT‐BMT information. Importantly, BIPQ, PHQ‐9, and GAD‐7 total scores were similar between these two groups.

## 5 CONCLUSIONS

While HSCT continues to remain a promising form of treatment and advances in transplant medicine have led to reduced morbidity and mortality from HSCT, HRQOL remains an important consideration. Our study suggests that addressing possibly modifiable psychological risk factors prior to and/or during recovery from HSCT has the potential to improve this important aspect of patient experience during transplant and also health outcomes.

## AUTHOR CONTRIBUTIONS

**Steven C. Ames:** Conceptualization (lead); investigation (lead); methodology (lead); project administration (lead); writing – original draft (lead); writing – review and editing (lead). **Lori Lange:** Conceptualization (supporting); methodology (supporting); writing – original draft (supporting); writing – review and editing (supporting). **Gretchen Ames:** Conceptualization (supporting); investigation (supporting); writing – original draft (supporting); writing – review and editing (supporting). **Michael Heckman:** Data curation (lead); formal analysis (lead); methodology (supporting); writing – original draft (supporting); writing – review and editing (supporting). **Launia J. White:** Data curation (lead); formal analysis (lead). **Vivek Roy:** Conceptualization (supporting); methodology (supporting); writing – review and editing (supporting). **James M. Foran:** Conceptualization (supporting); methodology (supporting); writing – review and editing (supporting).

## CONFLICT OF INTEREST STATEMENT

The authors declare no competing interests. This research was not supported by any research funding.

## Supporting information


Figure S1.
Table S1.
Table S2.
Table S3.

## Data Availability

The data that support the findings of this study are available upon reasonable request from the corresponding author.

## References

[1] Bazinet A , Popradi G . A general practitioner's guide to hematopoietic stem‐cell transplantation. Curr Oncol. 2019;26(3):187‐191. 31285665 10.3747/co.26.5033 PMC6588058

[2] Balassa K , Danby R , Rocha V . Haematopoietic stem cell transplants: principles and indications. Br J Hosp Med (Lond). 2019;80(1):33‐39. 30592675 10.12968/hmed.2019.80.1.33

[3] Janicsak H , Ungvari GS , Gazdag G . Psychosocial aspects of hematopoietic stem cell transplantation. World J Transplant. 2021;11(7):263‐276. 34316451 10.5500/wjt.v11.i7.263 PMC8290998

[4] Amonoo HL , Massey CN , Freedman ME , et al. Psychological considerations in hematopoietic stem cell transplantation. Psychosomatics. 2019;60(4):331‐342. 31072626 10.1016/j.psym.2019.02.004 PMC6626677

[5] Majhail NS . Long‐term complications after hematopoietic cell transplantation. Hematol Oncol Stem Cell Ther. 2017;10(4):220‐227. 28641097 10.1016/j.hemonc.2017.05.009 PMC5925745

[6] Hierlmeier S , Eyrich M , Wolfl M , Schlegel PG , Wiegering V . Early and late complications following hematopoietic stem cell transplantation in pediatric patients–a retrospective analysis over 11 years. PloS One. 2018;13(10):e0204914. 30325953 10.1371/journal.pone.0204914 PMC6191171

[7] Trask PC , Paterson A , Riba M , et al. Assessment of psychological distress in prospective bone marrow transplant patients. Bone Marrow Transplant. 2002;29(11):917‐925. 12080358 10.1038/sj.bmt.1703557

[8] Grulke N , Albani C , Bailer H . Quality of life in patients before and after haematopoietic stem cell transplantation measured with the European Organization for Research and Treatment of cancer (EORTC) Quality Of Life Core Questionnaire QLQ‐C30. Bone Marrow Transplant. 2012;47(4):473‐482. 21602898 10.1038/bmt.2011.107

[9] Liang Y , Wang H , Niu M , Zhu X , Cai J , Wang X . Longitudinal analysis of the relationships between social support and health‐related quality of life in hematopoietic stem cell transplant recipients. Cancer Nurs. 2019;42(3):251‐257. 29933311 10.1097/NCC.0000000000000616

[10] Brice L , Gilroy N , Dyer G , et al. Predictors of quality of life in allogeneic hematopoietic stem cell transplantation survivors. J Psychosoc Oncol. 2021;39(4):534‐552. 33468039 10.1080/07347332.2020.1870644

[11] Leventhal H , Nerenz DR , Steele DJ . Illness representations and coping with health threats. In: Baum A , Taylor SE , Singer JE , eds. Handbook of Psychology and Health, Volume IV: Social Psychological Aspects of Health. Erlbaum; 1984:219‐252.

[12] Hagger MS , Koch S , Chatzisarantis NLD , Orbell S . The common sense model of self‐regulation: meta‐analysis and test of a process model. Psychol Bull. 2017;143(11):1117‐1154. 28805401 10.1037/bul0000118

[13] Dempster M , Howell D , McCorry NK . Illness perceptions and coping in physical health conditions: a meta‐analysis. J Psychosom Res. 2015;79(6):506‐513. 26541550 10.1016/j.jpsychores.2015.10.006

[14] Nelson AM , Juckett MB , Coe CL , Costanzo ES . Illness perceptions predict health practices and mental health following hematopoietic stem cell transplantation. Psychooncology. 2019;28(6):1252‐1260. 30942921 10.1002/pon.5075 PMC6546544

[15] Pillay B , Lee SJ , Katona L , Burney S , Avery S . Psychosocial factors associated with quality of life in allogeneic stem cell transplant patients prior to transplant. Psychooncology. 2014;23(6):642‐649. 24375571 10.1002/pon.3462

[16] Pillay B , Lee SJ , Katona L , De Bono S , Burney S , Avery S . A prospective study of the relationship between sense of coherence, depression, anxiety, and quality of life of haematopoietic stem cell transplant patients over time. Psychooncology. 2015;24(2):220‐227. 25052297 10.1002/pon.3633

[17] Elwadhi D , Khandelwal SK , Kumar L , Sharma A . Short‐term impact of hematopoietic stem cell transplantation on psychiatric morbidity and quality of life in hematological malignancies in adults. Indian J Psychol Med. 2020;42(1):61‐68. 31997867 10.4103/IJPSYM.IJPSYM_70_19 PMC6970306

[18] Hjermstad MJ , Loge JH , Evensen SA , Kvaloy SO , Fayers PM , Kaasa S . The course of anxiety and depression during the first year after allogeneic or autologous stem cell transplantation. Bone Marrow Transplant. 1999;24(11):1219‐1228. 10642812 10.1038/sj.bmt.1702046

[19] Felder‐Puig R , di Gallo A , Waldenmair M , et al. Health‐related quality of life of pediatric patients receiving allogeneic stem cell or bone marrow transplantation: results of a longitudinal, multi‐center study. Bone Marrow Transplant. 2006;38(2):119‐126. 16820782 10.1038/sj.bmt.1705417

[20] Hjermstad MJ , Knobel H , Brinch L , et al. A prospective study of health‐related quality of life, fatigue, anxiety and depression 3‐5 years after stem cell transplantation. Bone Marrow Transplant. 2004;34(3):257‐266. 15170167 10.1038/sj.bmt.1704561

[21] D'Souza A , Brazauskas R , Stadtmauer EA , et al. Trajectories of quality of life recovery and symptom burden after autologous hematopoietic cell transplantation in multiple myeloma. Am J Hematol. 2023;98(1):140‐147. 35567778 10.1002/ajh.26596 PMC9659666

[22] Spitzer RL , Kroenke K , Williams JB . Validation and utility of a self‐report version of PRIME‐MD: the PHQ primary care study. Primary care evaluation of mental disorders. Patient health questionnaire. Jama. 1999;282(18):1737‐1744. 10568646 10.1001/jama.282.18.1737

[23] Kroenke K , Spitzer RL , Williams JB . The PHQ‐9: validity of a brief depression severity measure. J Gen Intern Med. 2001;16(9):606‐613. 11556941 10.1046/j.1525-1497.2001.016009606.x PMC1495268

[24] Spitzer RL , Kroenke K , Williams JB , Lowe B . A brief measure for assessing generalized anxiety disorder: the GAD‐7. Arch Intern Med. 2006;166(10):1092‐1097. 16717171 10.1001/archinte.166.10.1092

[25] Lowe B , Decker O , Muller S , et al. Validation and standardization of the generalized anxiety disorder screener (GAD‐7) in the general population. Med Care. 2008;46(3):266‐274. 18388841 10.1097/MLR.0b013e318160d093

[26] Broadbent E , Petrie KJ , Main J , Weinman J . The brief illness perception questionnaire. J Psychosom Res. 2006;60(6):631‐637. 16731240 10.1016/j.jpsychores.2005.10.020

[27] van Lunteren M , Landewe R , Fongen C , Ramonda R , van der Heijde D , van Gaalen FA . Do illness perceptions and coping strategies change over time in patients recently diagnosed with axial spondyloarthritis? J Rheumatol. 2020;47(12):1752‐1759. 32414957 10.3899/jrheum.191353

[28] Damman W , Liu R , Kaptein AA , et al. Illness perceptions and their association with 2 year functional status and change in patients with hand osteoarthritis. Rheumatology (Oxford). 2018;57(12):2190‐2199. 30107461 10.1093/rheumatology/key231

[29] McQuellon RP , Russell GB , Cella DF , et al. Quality of life measurement in bone marrow transplantation: development of the functional assessment of cancer therapy‐bone marrow transplant (FACT‐BMT) scale. Bone Marrow Transplant. 1997;19(4):357‐368. 9051246 10.1038/sj.bmt.1700672

[30] Braamse AM , Gerrits MM , van Meijel B , et al. Predictors of health‐related quality of life in patients treated with auto‐ and Allo‐SCT for hematological malignancies. Bone Marrow Transplant. 2012;47(6):757‐769. 21725373 10.1038/bmt.2011.130

[31] Richardson DR , Huang Y , McGinty HL , et al. Psychosocial risk predicts high readmission rates for hematopoietic cell transplant recipients. Bone Marrow Transplant. 2018;53(11):1418‐1427. 29445123 10.1038/s41409-018-0118-4 PMC6092254

[32] Kroemeke A , Sobczyk‐Kruszelnicka M , Kwissa‐Gajewska Z . Everyday life following hematopoietic stem cell transplantation: decline in physical symptoms within the first month and change‐related predictors. Qual Life Res. 2018;27(1):125‐135. 28900828 10.1007/s11136-017-1705-3 PMC5770502

[33] Pillay B , Lee SJ , Katona L , Burney S , Avery S . Psychosocial factors predicting survival after allogeneic stem cell transplant. Support Care Cancer. 2014;22(9):2547‐2555. 24736876 10.1007/s00520-014-2239-7

[34] Rentscher KE , Carroll JE , Juckett MB , et al. Sleep disruption, fatigue, and depression as predictors of 6‐year clinical outcomes following allogeneic hematopoietic cell transplantation. J Natl Cancer Inst. 2021;113(10):1405‐1414. 33693799 10.1093/jnci/djab032 PMC8633423

[35] Saunders IM , Tan M , Koura D , Young R . Long‐term follow‐up of hematopoietic stem cell transplant survivors: a focus on screening, monitoring, and therapeutics. Pharmacotherapy. 2020;40(8):808‐841. 32652612 10.1002/phar.2443

[36] Amonoo HL , El‐Jawahri A , Celano CM , et al. A positive psychology intervention to promote health outcomes in hematopoietic stem cell transplantation: the PATH proof‐of‐concept trial. Bone Marrow Transplant. 2021;56(9):2276‐2279. 33879852 10.1038/s41409-021-01296-9 PMC8416696

[37] El‐Jawahri A , LeBlanc T , VanDusen H , et al. Effect of inpatient palliative care on quality of life 2 weeks after hematopoietic stem cell transplantation: a randomized clinical trial. JAMA. 2016;316(20):2094‐2103. 27893130 10.1001/jama.2016.16786 PMC5421101

[38] El‐Jawahri A , Traeger L , Greer JA , et al. Effect of inpatient palliative care during hematopoietic stem‐cell transplant on psychological distress 6 months after transplant: results of a randomized clinical trial. J Clin Oncol. 2017;35(32):3714‐3721. 28926288 10.1200/JCO.2017.73.2800 PMC5675739

[39] Chakraborty R , Hamilton BK , Hashmi SK , Kumar SK , Majhail NS . Health‐related quality of life after autologous stem cell transplantation for multiple myeloma. Biol Blood Marrow Transplant. 2018;24(8):1546‐1553. 29626515 10.1016/j.bbmt.2018.03.027 PMC6108907

