# Supplementary material for: A prospective study of the relationship between illness perception, depression, anxiety, and quality of life in hematopoietic stem cell transplant patients
Source: Cancer Med. 2024 Jan 11;13(3):e6906. doi: 10.1002/cam4.6906 (PMC10905249; doi:10.1002/cam4.6906)

**Supplemental Table 1: Number of FACT-BMT questions answered at the day 100 and 1-year time points**

| Number of FACT-BMT questions answered (of 37 total) | No. (%) of patients (N=205) |
| --- | --- |
| FACT-BMT at day 100 |  |
| 0 questions answered | 49 (23.9%) |
| 23 questions answered | 1 (0.5%) |
| 27 questions answered | 1 (0.5%) |
| 32 questions answered | 1 (0.5%) |
| 33 questions answered | 1 (0.5%) |
| 34 questions answered | 4 (2.0%) |
| 35 questions answered | 11 (5.4%) |
| 36 questions answered | 40 (19.5%) |
| 37 questions answered | 97 (47.3%) |
| FACT-BMT at 1-year |  |
| 0 questions answered | 64 (31.2%) |
| 27 questions answered | 2 (1.0%) |
| 33 questions answered | 1 (0.5%) |
| 34 questions answered | 2 (1.0%) |
| 35 questions answered | 7 (3.4%) |
| 36 questions answered | 39 (19.0%) |
| 37 questions answered | 90 (43.9%) |

**Supplemental Table 2: Summary of baseline BIPQ individual questions prior to any reverse scoring**

| BIPQ question | Mean (Standard deviation) | Median (Range) | Interquartile range |
| --- | --- | --- | --- |
| 1. How much does your illness affect your life? (0=no affect at all, 10=severely affects my life) | 6.0 (2.7) | 6 (0 – 10) | 4 - 8 |
| 2. How long do you think your illness will continue? (0=a very short time, 10=forever) | 4.3 (3.3) | 5 (0 – 10) | 3 - 8 |
| 3. How much control do you feel you have over your illness? (0=absolutely no control, 10=extreme amount of control) | 4.9 (2.9) | 5 (0 – 10) | 3 – 7 |
| 4. How much do you think your treatment can help your illness? (0=not at all, 10=extremely helpful) | 9.2 (1.3) | 10 (4 – 10) | 9 – 10 |
| 5. How much do you experience symptoms from your illness? (0=no symptoms at all, 10=many severe symptoms) | 4.5 (2.7) | 5 (0 – 10) | 2 – 7 |
| 6. How concerned are you about your illness? (0=not at all concerned, 10=extremely concerned) | 7.4 (2.8) | 8 (0 – 10) | 5 – 10 |
| 7. How well do you feel you understand your illness? (0=don’t understand at all, 10=understand very clearly) | 8.5 (1.5) | 9 (2 – 10) | 8 - 10 |
| 8. How much does your illness affect you emotionally? (e.g. does it make you angry, scared, upset or depressed?) (0=not at all affected emotionally, 10=extremely affected emotionally) | 4.2 (2.8) | 4 (0 – 10) | 2 - 7 |

**Supplemental Table 3: Comparison of characteristics between patients with and without a FACT-BMT total score at either day 100 or 1 year**

|  | FACT-BMT at either day 100 or 1 year (N=165) | | No FACT-BMT at either day 100 or 1 year (N=40) | |  |
| --- | --- | --- | --- | --- | --- |
| Variable | N | Median (minimum, maximum) or No. (%) of patients | N | Median (minimum, maximum) or No. (%) of patients | P-value |
| Age at transplant (years) | 165 | 60 (22, 80) | 40 | 60 (22, 74) | 0.18 |
| Sex (Male) | 165 | 86 (52.1%) | 40 | 21 (52.5%) | 1.00 |
| Ethnicity (Hispanic/Latino) | 164 | 8 (4.9%) | 40 | 3 (7.5%) | 0.45 |
| Race | 163 |  | 40 |  | 0.82 |
| White |  | 143 (87.7%) |  | 34 (85.0%) |  |
| Black or African American |  | 16 (9.8%) |  | 5 (12.5%) |  |
| Other |  | 4 (2.5%) |  | 1 (2.5%) |  |
| Marital status | 165 |  | 40 |  | 0.33 |
| Never married |  | 10 (6.1%) |  | 2 (5.0%) |  |
| Currently married |  | 123 (74.5%) |  | 28 (70.0%) |  |
| Separated |  | 3 (1.8%) |  | 1 (2.5%) |  |
| Divorced |  | 15 (9.1%) |  | 8 (20.0%) |  |
| Widowed |  | 6 (3.6%) |  | 1 (2.5%) |  |
| Cohabitating with partner |  | 8 (4.8%) |  | 0 (0.0%) |  |
| Level of school completed | 165 |  | 39 |  | 0.35 |
| Some high school or less |  | 9 (5.5%) |  | 3 (7.7%) |  |
| High school/GED |  | 36 (21.8%) |  | 7 (17.9%) |  |
| Some college/associate/technical |  | 42 (25.5%) |  | 16 (41.0%) |  |
| College graduate |  | 52 (31.5%) |  | 9 (23.1%) |  |
| Graduate school |  | 26 (15.8%) |  | 4 (10.3%) |  |
| Current employment status | 165 |  | 40 |  | 0.32 |
| Employed |  | 63 (38.2%) |  | 17 (42.5%) |  |
| On leave |  | 27 (16.4%) |  | 5 (12.5%) |  |
| Retired |  | 48 (29.1%) |  | 7 (17.5%) |  |
| Disabled |  | 17 (10.3%) |  | 8 (20.0%) |  |
| Unemployed |  | 10 (6.1%) |  | 3 (7.5%) |  |
| Annual household gross income | 160 |  | 38 |  | 0.70 |
| Less than $20,000 |  | 10 (6.3%) |  | 4 (10.5%) |  |
| $20,000-$39,999 |  | 25 (15.6%) |  | 3 (7.9%) |  |
| $40,000-$59,999 |  | 23 (14.4%) |  | 6 (15.8%) |  |
| $60,000-$79,999 |  | 31 (19.4%) |  | 8 (21.1%) |  |
| $80,000-$99,999 |  | 29 (18.1%) |  | 5 (13.2%) |  |
| $100,000 or more |  | 42 (26.3%) |  | 12 (31.6%) |  |
| Smoking history | 164 |  | 40 |  | 1.00 |
| Never smoked |  | 89 (54.3%) |  | 22 (55.0%) |  |
| Past smoker |  | 68 (41.5%) |  | 16 (40.0%) |  |
| Current smoker |  | 7 (4.3%) |  | 2 (5.0%) |  |
| Illicit drug use |  |  |  |  |  |
| Any illicit drug use | 165 | 9 (5.5%) | 40 | 4 (10.0%) | 0.29 |
| Marijuana | 165 | 9 (5.5%) | 40 | 3 (7.5%) | 0.71 |
| Prescription drugs | 165 | 0 (0.0%) | 40 | 1 (2.5%) | 0.20 |
| Primary disease type | 165 |  | 40 |  | <0.001 |
| Acute lymphocytic leukemia |  | 7 (4.2%) |  | 5 (12.5%) |  |
| Acute myeloid leukemia |  | 23 (13.9%) |  | 2 (5.0%) |  |
| Other acute leukemia |  | 2 (1.2%) |  | 0 (0.0%) |  |
| Chronic myelogenous leukemia |  | 1 (0.6%) |  | 4 (10.0%) |  |
| Myelodysplastic syndrome/disorder |  | 14 (8.5%) |  | 8 (20.0%) |  |
| Hodgkin’s disease |  | 7 (4.2%) |  | 5 (12.5%) |  |
| Non Hodgkins lymphoma |  | 24 (14.5%) |  | 6 (15.0%) |  |
| Plasma cell disease |  | 83 (50.3%) |  | 7 (17.5%) |  |
| Aplastic anemia |  | 1 (0.6%) |  | 0 (0.0%) |  |
| Chronic lymphocytic leukemia |  | 1 (0.6%) |  | 1 (2.5%) |  |
| Other |  | 2 (1.2%) |  | 2 (5.0%) |  |
| Alcohol history | 164 |  | 40 |  | 0.84 |
| Never |  | 76 (46.3%) |  | 20 (50.0%) |  |
| Monthly or less |  | 45 (27.4%) |  | 13 (32.5%) |  |
| 2 to 4 times a month |  | 23 (14.0%) |  | 5 (12.5%) |  |
| 2 to 3 times a week |  | 12 (7.3%) |  | 1 (2.5%) |  |
| 4 or more times a week |  | 8 (4.9%) |  | 1 (2.5%) |  |
| Body mass index | 161 | 28.3 (0.0, 44.6) | 39 | 29.0 (18.3, 55.6) | 0.057 |
| Type of transplant | 165 |  | 40 |  | 0.002 |
| Autologous |  | 111 (67.3%) |  | 16 (40.0%) |  |
| Allogeneic |  | 54 (32.7%) |  | 24 (60.0%) |  |
| BIPQ total score (baseline) | 164 | 35 (5, 62) | 39 | 35 (12, 48) | 0.78 |
| PHQ-9 total score (baseline) | 165 | 3 (0, 21) | 40 | 3 (0, 14) | 0.85 |
| GAD-7 total score (baseline) | 165 | 2 (0, 21) | 40 | 1 (0, 19) | 0.74 |
| P-values result from a Wilcoxon rank sum test (continuous and ordinal variables) or Fisher’s exact test (categorical variables). | | | | | |

**Supplemental Figure 1: Histograms of total scores on psychological factors for participants receiving autologous and allogeneic stem cell transplant**


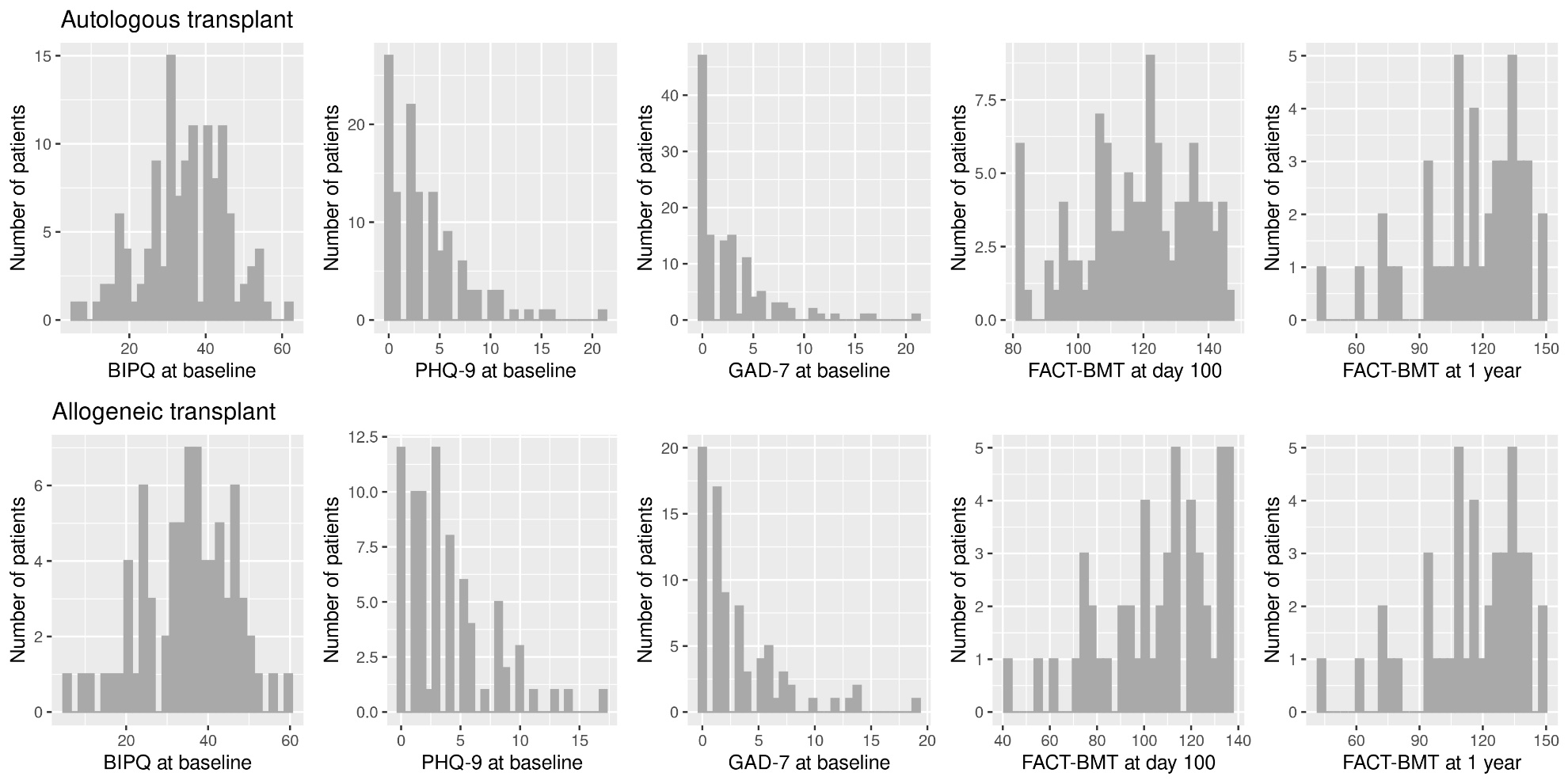

Supplement: Supplementary file 1 — Figure S1. Table S1. Table S2. Table S3. [file CAM4-13-e6906-s001.docx]
